# Patient-specific implants combined with 3D-printed drilling guides for corrective osteotomies of multiplanar tibial and femoral shaft malunions leads to more accurate corrections

**DOI:** 10.1007/s00068-024-02755-w

**Published:** 2025-01-24

**Authors:** M. G. E. Oldhoff, C. Posada Alvarez, K. Ten Duis, J. N. Doornberg, N. Assink, F. F. A. IJpma

**Affiliations:** 1 Department of Trauma Surgery, University Medical Centre Groningen, University of Groningen, Groningen, The Netherlands; 2 3D Lab, University Medical Centre Groningen, University of Groningen, Groningen, The Netherlands; 3 Department of Orthopaedic Surgery, University Medical Center Groningen, University of Groningen, Groningen, The Netherlands; 4 Department of Orthopaedic Trauma Surgery, Flinders Medical Centre, Adelaide, Australia

**Keywords:** 3D technology, Corrective osteotomy, Tibial shaft, Femoral shaft, Patient-specific instrumentation, Virtual surgical planning

## Abstract

**Purpose:**

The aim of this study was to evaluate the feasibility of using patient-specific implants (PSI) for complex shaft corrective osteotomies in multiplanar deformities of long bones in the lower extremities. Additionally, it aimed to investigate the added value of these implants by quantifying surgical accuracy on postoperative CT, comparing their outcomes to two commonly used techniques: 3D virtual visualizations and 3D-printed surgical guides.

**Methods:**

Six tibial and femoral shaft corrective osteotomies were planned and performed on three Thiel embalmed human specimen. Depending on the specimen a different respective technique was used; 1) ‘3D Visualization’ using 3D virtual plan preoperatively and free-hand corrective osteotomy techniques with standard manually contoured plates; 2) ‘3D guided’ utilizing 3D surgical guides and manually contouring of conventional implant; and 3)*‘*3D PSI’ utilizing a 3D surgical guide with a patient-specific implant. Accuracy of the corrections was assessed through measurements for varus/valgus angulation, ante/recurvation, rotation and osteotomy plane error as quantified on postoperative CT-scans.

**Results:**

Twelve corrective osteotomies were performed. For, the median difference between the surgical plan and postoperative CT assessment was 3.4°, 4.6°, and 2.2° for the ‘3D visualization’, ‘3D guided’, and ‘3D PSI’ methods respectively. Regarding ante/recurvation, the differences were 3.8°, 43.8°, and 1.2°, respectively. For rotation, the differences were 11.9°, 18.7°, and 3.5°, respectively. Discrepancies between planned and executed levels of osteotomy plane were 6.2 mm, 3.2 mm, and 1.4 mm, respectively.

**Conclusion:**

PSIs with 3D-printed drilling guides for complex multiplanar corrective osteotomies of femoral and tibial shaft malunions is feasible and achieves accurate corrections. This technique enables precise determination of the osteotomy plane, guides correction in all three planes, and ensures satisfactory implant fitting; thus accurately translating the virtual surgical plan into clinical practice. The 3D PSI method is beneficial for complex cases with significant multiplanar deformities in bone anatomy, particularly with rotational malalignment.

## Introduction

Malunion of the femur or tibial shaft may occur due to secondary displacement in a cast or suboptimal fracture reduction during operative treatment. Rotational control is especially challenging with intramedullary nailing in case of closed fracture reduction of comminuted fractures [1], and reported values of incidence for malrotation are 15–40% of femoral fractures and 20–40% of tibial fractures treated with intramedullary fixation [2–4]. Malalignment may lead to a range of complaints, including altered gait patterns, muscle fatigue, chronic pain, and aesthetic concerns, significantly impacting a patient’s physical function and quality of life [5, 6]. Moreover, abnormal loads placed on the joints due to malalignment can cause degenerative joint disease, leading to further discomfort and potential conversion to joint replacement surgery [7, 8].

Treatment of malunited bones usually consist of corrective osteotomy surgery. Osteotomy for long bone deformities involves surgically cutting and realigning the bone to correct malalignment. Traditionally surgeons use two-dimensional (2D) X-ray images to plan the surgery and then perform freehand osteotomies using K-wires and a goniometer to realign the bone, resulting in unpredictable outcomes [9–11]. Currently, three dimensional (3D) technology is increasingly used for corrective osteotomy surgery [12]. It utilizes computed tomography (CT) data to generate 3D visualization of malunions, enabling precise quantification of deformations in all three dimensions. Additionally, it facilitates the creation of a preoperative 3D virtual surgical plan. Surgical guides can subsequently be utilized to translate the virtual plan to the operation room. These surgical guides assist the surgeon with the osteotomy and repositioning of the bone, leading to more accurate and predictable outcomes [9, 13–17]. Moreover, secondary callus formation resulting from malunions, and potential large correctional angles may lead to improper fit of conventional implants, even after manual bending. In such cases, the 3D workflow can be further extended with the use of patient-specific implants (PSI). These implants are tailored to patient’s anatomy, ensuring proper fit to guide bone realignment and provide optimal fixation [18–20]. Thus, making these suitable for a variety of corrective osteotomy procedures including femoral and tibial shaft corrections [21–25] and might possibly lead to more accurate corrective osteotomies and improving clinical outcomes.

This human specimen study aimed to evaluate the feasibility of using PSIs for complex corrective osteotomies of the shaft of long bones in the lower extremities. Due to its proof-of-principle nature, the study conducted using a limited number of specimens, focusing on demonstrating feasibility rather than extensive validation. This feasibility study is a necessary step under the MDR, forming part of the technical documentation required prior to clinical application. Additionally, this study aimed to investigate the added value of these customized implants by quantifying surgical accuracy on postoperative computed tomography and comparing their postoperative outcomes to two commonly used techniques: 3D virtual visualizations and 3D-printed surgical guides.

## Materials and methods

### Specimens

This study serves as a proof of principle, conducted using only three cadaver specimens to investigate the three different techniques for both long bones of the lower extremity. Our focus was solely on evaluating the accuracy, without consideration of clinical outcomes. Three full-body Thiel embalmed human cadaver specimens were used [26]. Each specimen underwent the same corrective osteotomy surgery of all femurs and tibias to enable direct comparison. In each cadaver, a pre-experiment, as well as post-surgical correction-, CT scan of the lower extremities was obtained that included the entire femurs and tibias according to our standard clinical imaging protocol (0.6 mm slice thickness, voxel size 0.4 mm). CT data served as base for our 3D surgical planning and evaluation.

### Study setup

**Fig. 1:**
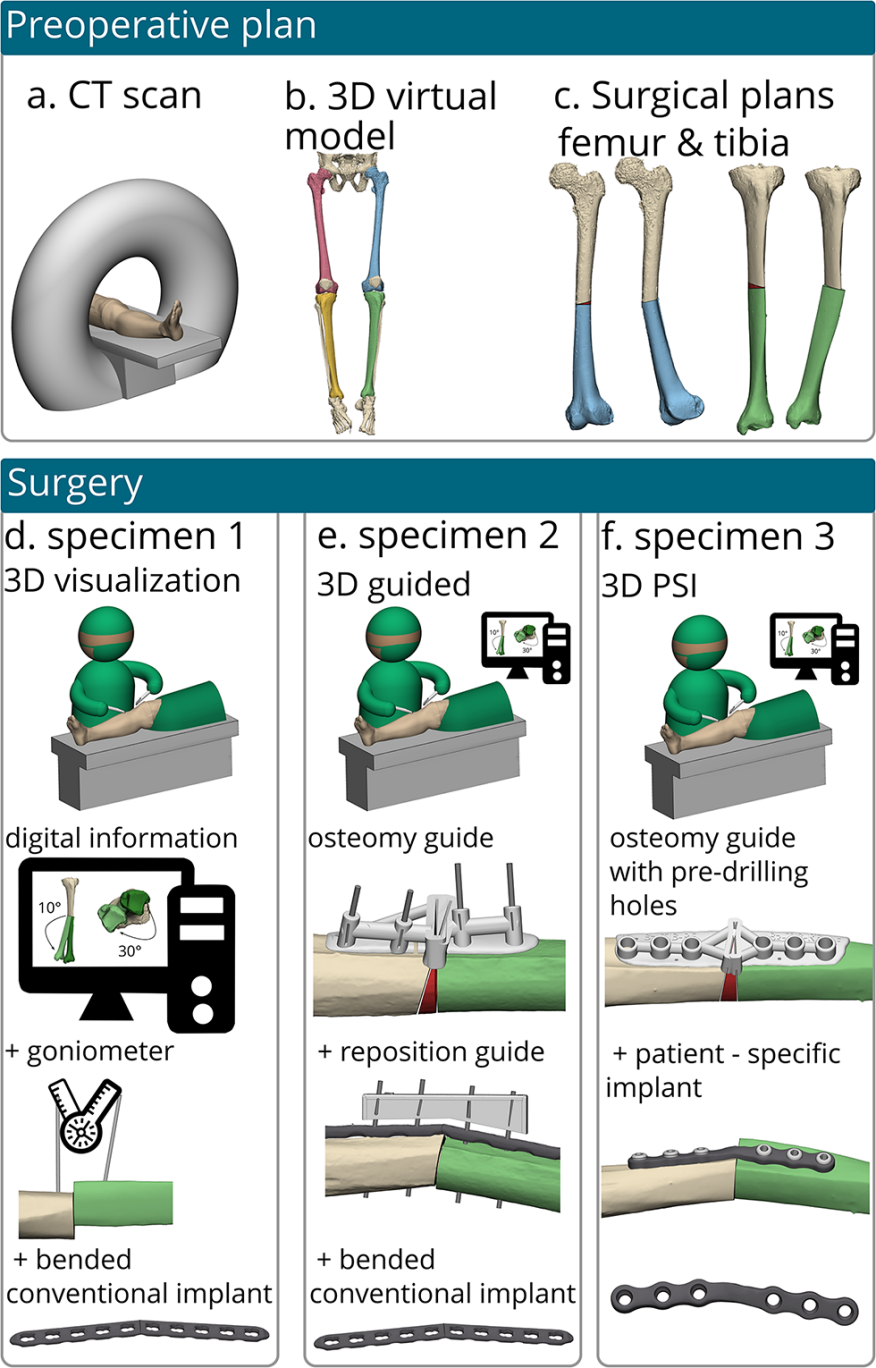
Schematic workflows for corrective osteotomy surgeries of the femur and tibia in a cadaveric study. It shows the preoperative planning stages (**a-c**) alongside with the three procedural techniques used: **‘3D visualization’** utilizing traditional on-screen visualization of the virtual surgery plan, free-hand repositioning of the bone and a contoured conventional implant (**d**), **‘3D guided’** employing an 3D-printed osteotomy guide, reposition guide and a contoured conventional implant (**e**), and **‘3D PSI’** integrating an osteotomy guide with pre-drilled holes and a PSI, specially designed and produced within 5 days for performing the corrective osteotomy (**f**)

As the human cadaveric specimens lacked deformities, traditional corrective osteotomies to achieve an anatomical state were unfeasible. Thus, an alternative experimental setup involving corrective osteotomies in a reversed direction (i.e. transforming normal anatomy into malposition) was performed to evaluate three different techniques for corrective osteotomy surgery: (1) using 3D virtual plan preoperatively and merely traditional 2D imaging techniques and a goniometer with standard manually contoured implants perioperative (i.e. 3D visualization); (2) with 3D surgical guides and manually contouring of conventional implant during the surgery (i.e. 3D guided); and (3) with 3D surgical guides in combination with a PSI (i.e. 3D PSI). Malpositions of the femur and tibia were introduced by simulating virtual osteotomies and altering the distal part in various orientations (see Fig. 1c). A total of twelve bones underwent (reversed) corrective osteotomy surgery: six femurs and six tibias. Corrective osteotomies were virtually planned for all bones to achieve a similar deformity with the same degree of translation and rotation for every specimen. The femur osteotomy was planned in the middle of the shaft, after osteotomy the distal part was first moved 10° in valgus, followed by 10° in recurvation and finally 30° in endo-rotation. The tibias were virtually planned to achieve a similar corrective osteotomy; after osteotomy located at the middle of the shaft, the distal part was first moved 10° in varus, followed by 10° in recurvation and finally 30° in exo-rotation.

### 3D modelling

First step entailed segmenting CT data using Mimics Medical software (version 26.0, Materialise, Leuven, Belgium) to generate 3D models of all bones in the lower extremities (see Fig. 1b). A predetermined threshold value for bone (≥ 226 Hounsfield Units) was utilized to select the bone region. Subsequently, femur and tibia were separated from adjacent bones using the split mask and edit mask tools. This left us with virtual 3D models of the bones of interest. These virtual 3D models were then imported into 3-Matic software (version 18.0, Materialise, Leuven, Belgium). Design tools were used to manually cut the bone, and the distal part of the bone was rotated in three directions (i.e. 10° valgus varus, 10° recurvation and 30° rotation). The 3D software 3-matic was used execute and design additional models or patient-specific instrumentation, such as the osteotomy guides with cutting slots and K-wire slots, the reposition guides with K-wire slots, the PSIs, and the anatomical models.

**Fig. 2:**
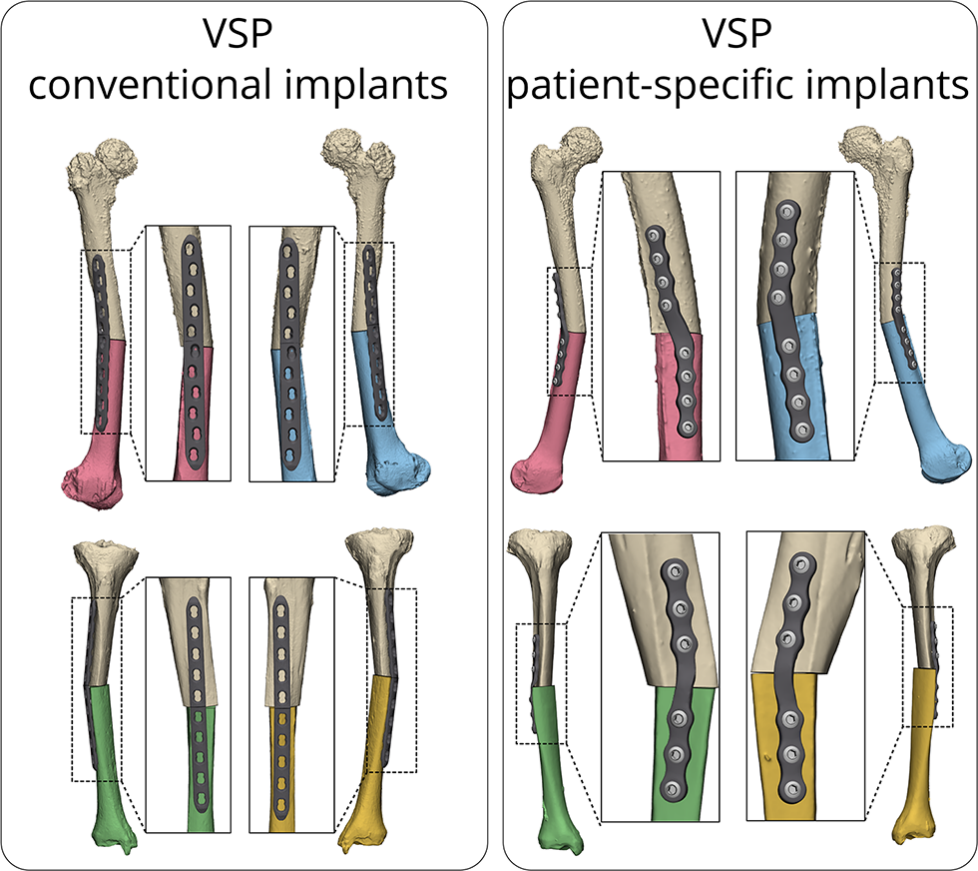
Virtual surgical plan (VSP) of femoral and tibial corrective osteotomy. The left side illustrates the 3D virtual plan of first and second specimen, ’3D visualization’ and ‘3D guided’, with conventional virtually contoured implants. The right side illustrates the 3D virtual plan of the third specimen, ‘3D PSI’, using PSIs

For ‘3D visualization’ method, no additional 3D-printed guides or 3D models were fabricated, only on-screen visualization of 3D virtual surgery plan (VSP) was allowed. For the ‘3D guided’ method two surgical guides per bone were fabricated; an osteotomy guide and a reposition guide. These guides were designed to fit the underlying bone and guide the osteotomy and subsequently ensuring correct alignment of the osteotomized bone ends according to the 3D surgical plan. The first guide, the osteotomy guide, includes a slot for the surgeon to perform the osteotomy and four slots for k-wire placement. The second guide, the reposition guide, consisted solely of the k-wire slots and could be positioned over the implant. Additionally, a 3D-printed hand-held model, representing the planned outcome, was utilized to manually pre-bend the conventional implants during surgery. Lastly the ‘3D PSI’ method consisted of one surgery guide and a PSI. The surgery guide was used for the osteotomy and pre-drilling of screws, while the implant was employed to hold the bone in the predetermined position. The implants were design using 3-Matic software (version 18.0, Materialise, Leuven, Belgium), Solidworks Professional software version 2020 (Dassault Systèmes Solidworks), and the Geomagic package for Solidworks (3D Systems). They were fabricated from medical-grade titanium alloy using a 5-axis milling machine at a regional company, Witec Medical B.V. (Stadskanaal, the Netherlands), within three days. All 3D VSPs with conventional implants and PSIs are illustrated in Fig. 2.

### Surgical procedure

For each femoral corrective osteotomy, the direct lateral subvastus approach was performed. For the tibia the antero-lateral approach was utilized. These surgical procedures were performed by trauma surgeons with more than 10 years of experience (KtD, JD, FIJ). The preplanned height of the incisions and osteotomies were verified during surgery using intraoperative fluoroscopy and a stainless-steel ruler. Prior to surgery, the 3D virtual surgical plans were discussed in a multidisciplinary meeting involving trauma surgeons, technical physicians, and engineers.

The first specimen, referred to as ‘3D visualization’, relied solely on 2D modalities throughout the procedure (Fig. 1d), with no utilization of 3D physical products. Two k-wires were positioned in parallel fashion, both distally and proximally to the intended osteotomy plane. Subsequently, a freehand osteotomy was performed, and a goniometer was employed to adjust the distal part with respect to the proximal end, ensuring the k-wires were positioned at the predetermined angle according to the planned correction. Following completion of the angle adjustments, fixation was achieved for both the distal as well as proximal ends using a large fragment Locking Compression Plates (LCP) for femur and tibia. The implants were bended intraoperatively and secured using 4.5 mm cortical screws.

The second specimen, ‘3D guided’, used two 3D-printed surgical guides during the procedure (Fig. 1e): an osteotomy guide and a reposition guide [23]. First, the osteotomy guide was positioned on the bone, and k-wires were drilled through designated holes. Subsequently, the osteotomy was performed through a slot in the guide. Upon removal of the osteotomy guide, the k-wires were placed in a parallel matter to realign proximal and distal bone ends according to preoperative planning. Next, implant and reposition guide were placed over the osteotomy site, and the implant was secured using 4.5 mm cortical screws. LCP implants were manually contoured pre-operatively using a 3D printed model of the desired shape of the anatomy.

The third specimen, ‘3D PSI’, utilized a 3D osteotomy guide along with a PSI (Fig. 1f). The osteotomy guide was positioned on the bone and secured with one or two k-wires. This guide featured an osteotomy slot and slots for drill sleeves, facilitating precise drilling and screw placement according to the preplanned directions and locations for the PSI. After removal of the guide, the implant was secured in place using cortical screws at the predrilled locations, thereby realigning the bone ends according to the predetermined plan due to the implants unique shape.

### Postoperative evaluations

After surgical procedures, each specimen underwent a postoperative CT-scan, using a slice thickness of 0.6 mm and with iterative metal artefact reduction techniques in order to quantify corrections. This CT scan was used to make a postoperative 3D model of the bones with the plate and screws. To assess the accuracy of the postoperative alignment, the distal bone parts of the femur or tibia were duplicated and aligned with the postoperative respective models. Measurements were taken to assess three-dimensional angulation, originating from the osteotomy site. These measurements were performed in 3D, encompassing all planes: (1) valgus/varus deviations; (2) ante/recurvation; and (3) rotation. In addition, height of performed osteotomy plane was verified.

### Statistical analysis

The overall dataset was divided in three groups: (1) 3D visualization; (2) 3D guided; and (3) 3D PSI. Accuracy of the continuous variables was summarized using median and interquartile range (IQR). Analysis included both left- and right tibia and femur. The achieved accuracy was assessed as the subtraction between the virtually planned 3D position and the obtained position postoperatively, evaluated in three dimensions with CTs, for (1) varus/valgus alignment; (2) ante/recurvation; (3) rotation; and (4) osteotomy plane error. To evaluate the differences between the three groups, a Kruskal-Wallis test was performed. Subsequently a Mann-Whitney U test was conducted to evaluate differences in the achieved accuracy between our focus group “3D PSI” and each of the other two respective groups separately, with a significance level of 0.05. Statistical analysis was performed using IBM SPSS Statistics (version 28.0.1.0, IBM, Chicago, IL, US). A post-hoc power analysis was performed to assess the statistical power of the study, based on the observed effect sizes and sample sizes.

## Results

**Fig. 3:**
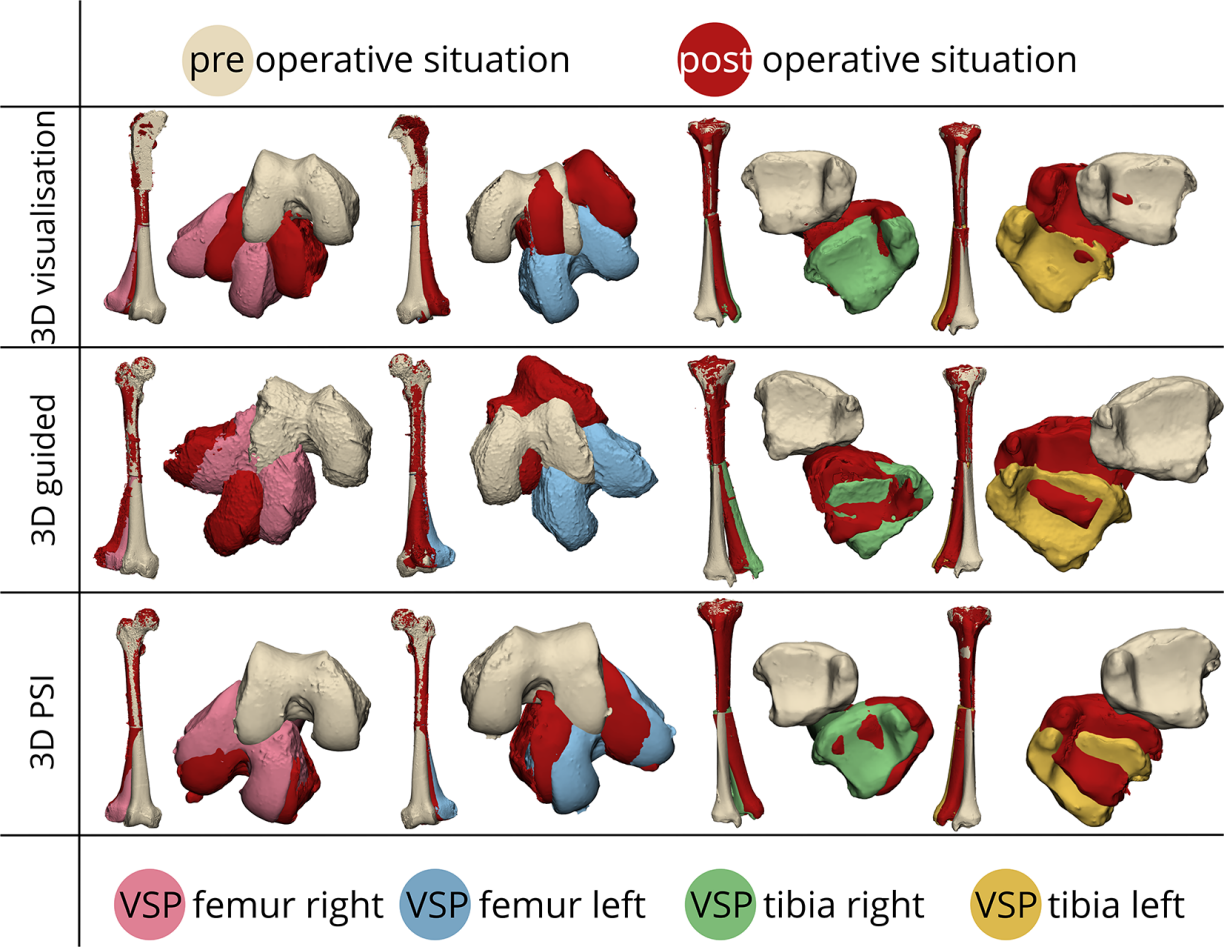
Visual representation and comparison between preoperative virtual surgical plan (VSP) and postoperative correction osteotomies. The beige model illustrates the preoperative situation, while the red models illustrate the achieved postoperative situations. The different colours in the distal bone parts indicate the 3D virtual surgical plans: pink for the right femur, blue for the left femur, green for the right tibia, and yellow for the left tibia. Each row showcases the implementation of three surgical techniques: 3D visualisation (top row), 3D guided (middle row), and 3D PSI (bottom row)

**Fig. 4:**
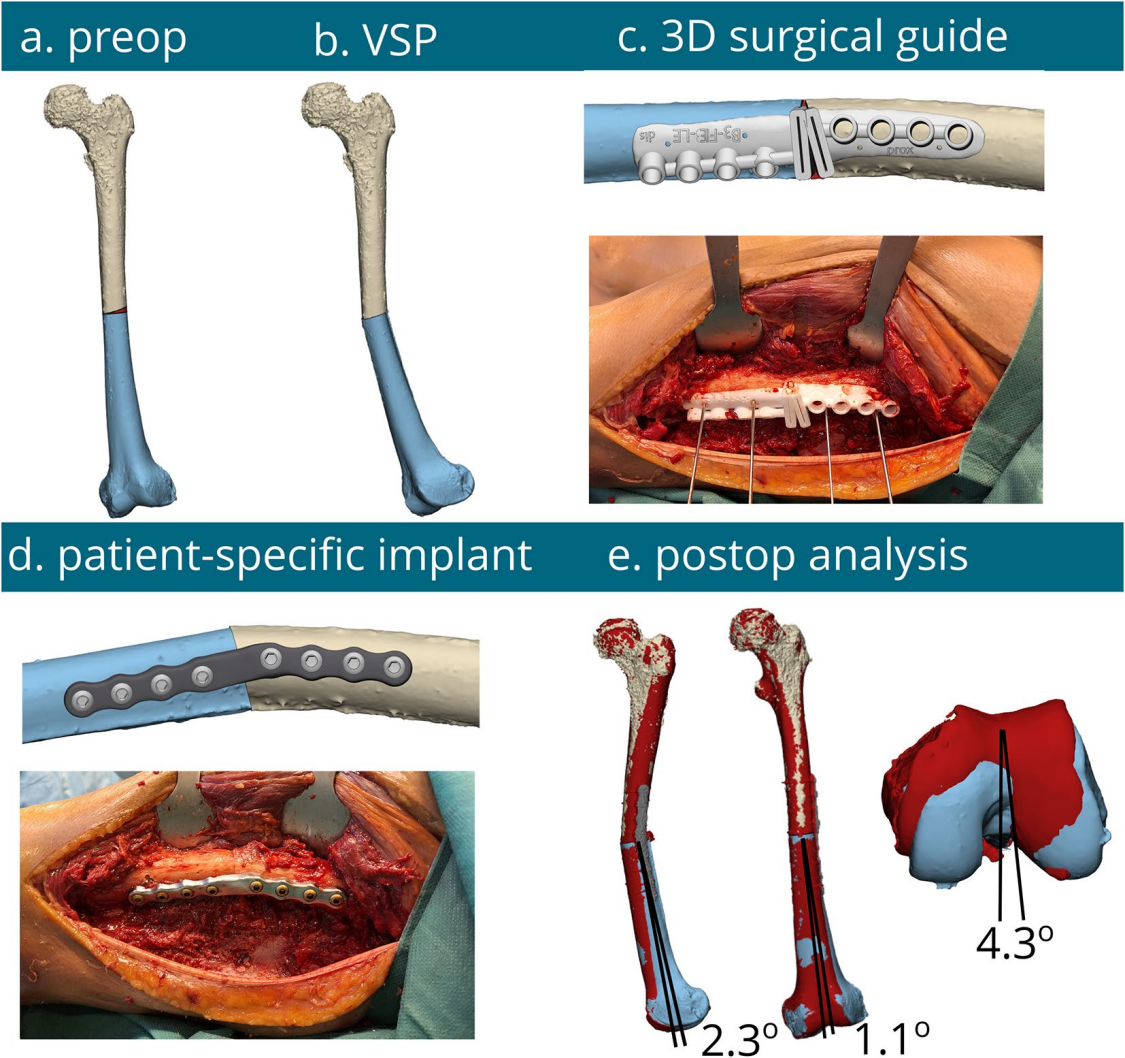
Surgical procedure of a multiplanar corrective osteotomy of the left femur using a PSI with a 3D-printed guide. From left to right: 3D model of the preoperative plan (**a**), the virtual surgery plan (VSP) of the correction (**b**), the surgical guide performing the osteotomy and predrilling of the screw holes (**c**), the milled titanium PSI (**d**) and the postoperative analysis with only 2.3⁰ varus/valgus, 1.1⁰ ante/recurvation and 4.3⁰ rotation difference between the preoperative virtual surgical plan (VSP) and postoperative CT evaluation (**e**)

Figure 3 provides a visual representation and comparison between preoperative virtual surgical plan (VSP) and postoperative correction osteotomies of all procedures, demonstrating that 3D-PSI (i.e. patient-specific implants with 3D-printed drilling guides) for complex multiplanar femoral and tibial shaft malunions are feasible and result in the most accurate corrections. Figure 4 illustrates the entire workflow of an multiplanar corrective osteotomy of the left femur using a PSI.

**Fig. 5:**
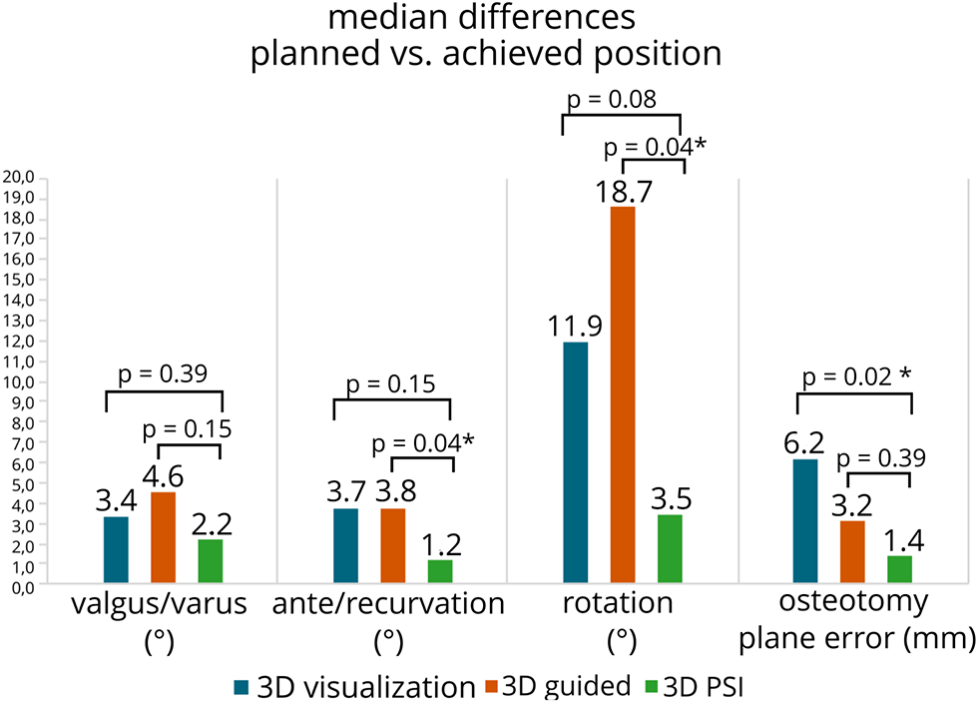
Postoperative evaluation of corrective osteotomies of both the femurs and tibias comparing three methods. Accuracy of the osteotomy was assessed based on the median difference between the 3D virtual surgical plan and the postoperative achieved position, considering the combined results of the femora and tibiae. The parameters used were varus/valgus angulation (degrees), ante/recurvation (degrees), rotation (degrees), and osteotomy plane error (millimeters). Overall, the most accurate correction was achieved using a 3D osteotomy guides combined with PSIs. Mann-Whitney U p-values are reported in the figure, * indicates significance

The Kruskal Willis test was performed to compare the three groups across multiple measures. No significant differences were found for valgus/varus (*p* = 0.368), ante/recurvation (*p* = 0.116), rotations (*p* = 0.069), or osteotomy plane error (*p* = 0.077). However, the Mann Whitney U test comparing the accuracy of the osteotomy between the “3D PSI” group and each of the other 2 methods individually revealed some significant differences (Fig. 5). As shown in Fig. 4, the achieved correction in the frontal plane (i.e. varus/valgus alignment), the median difference between the preoperative virtual surgical plan and postoperative CT evaluation of the ‘3D PSI’ group showed a median difference of 2.2° (IQR 1.8°-2.6°). The ‘3D visualization’ group had a median difference of 3.4° (IQR 1.8°-7.6°) (*p* = 0.39) and the ‘3D guided’ group had a median difference of 4.6° (IQR 2.3°-8.0°) (*p* = 0.15). In terms of ante/recurvation, the ‘3D PSI’ group had a median difference of 1.2° (IQR 0.6°-2.1), the ‘3D visualization’ group a median difference of 3.7° (IQR 1.6°-9.4°) (*p* = 0.15) and the ‘3D guide’ group a median difference of 3.8° (IQR 2.0°-13.3°) (*p* = 0.043). In terms of rotation (i.e. axial correction), the median difference for the ‘3D PSI’ group was 3.5° (IQR 1.5°-9.9°). for ‘3D visualization’ group the median difference of 11.9° (IQR 9.8°-16.6°) (*p* = 0.083) and the ‘3D guided’ group had a median difference was 18.7° (IQR 7.0°-23.7°) (*p* = 0.43). The median differences between the planned and achieved levels of the osteotomy planes were 1.4 mm (IQR 0.6–3.1 mm) for the ‘3D PSI’ group, the ‘3D visualization’ group had a median difference of 6.2 mm (IQR 4.9–8.3 mm) (*p* = 0.021) and the ‘3D guided’ group had a median difference of 3.2 mm (IQR 0.8–11.9 mm) (*p* = 0.39). Finally, A post hoc power analysis revealed that a sample size of three patients per group is required to detect a 10-degree difference in alignment (SD 4 degrees) with 80% power and an alpha of 0.05.

## Discussion

Corrective osteotomy surgeries of long bone posttraumatic deformities in the lower extremities can be technically challenging, requiring corrections in all three dimensions due to complex malunions. Callus formation and pathoanatomy can result in an unconventional fit of conventional (i.e. off-the-shelf) implants, complicating fixation and, consequently, accuracy of corrective osteotomy surgery. Although 3D virtual surgical planning and surgical guides have increasingly been used in corrective osteotomies to enhance accuracy and predictability of these procedures, the improper fit of conventional implants may still lead to unsatisfactory execution of the planned correction. Patient-specific implants (PSI), specially designed and produced for performing complex multiplanar corrective osteotomies, may provide helpful adjuncts in these cases: the focus of the current cadaveric study. This study demonstrated that the use of 3D PSIs in corrective osteotomy surgery of long bone malunions is feasible and preliminary results indicate that the PSI resulted in the most accurate translation of the virtual surgical plan to the operating room.

The PSI was designed, manufactured and applied within 5 days and achieved a good fit with the reconstruction bone, resulting in accurate surgical corrections within only +/- 3° deviation from the surgical plan. To the best of our knowledge, accuracy of PSI for correction of long bone malunions in the lower leg have not been reported in literature, to date. Several case reports and small series report the use of PSI for metaphyseal corrections of the tibial and femoral bones; Nkhwa et al. [27] report a case where PSI was used for a multiplanar femoral osteotomy in a paediatric patient. Due to the extensive deformity, a conventional implant was unsuitable, so a patient-specific plate was designed and successfully clinically applied to perform a corrective osteotomy of the distal femur, leading to accurate restoration of the mechanical axis on a postoperative x-ray. Studies regarding PSIs for corrective osteotomies of the proximal part of the tibia showed promising results as well [24, 25, 28]. Reported advantages included preplanning of screw and implant positions, plates tailored to fit the bone’s extensive deformities, and a reduced need for interoperative fluoroscopy images [25]. Our study extensively investigated patient-specific implants for multiplanar long bone shaft corrective osteotomy in the lower extremities. The customized implant’s precise fit ensured the bone segment was guided into the predetermined position, preventing secondary displacement after fixation, and proved to be more accurate and predictable than the other two methods studied. This innovative 3D workflow, along with the comparative nature of the study, adds to the current literature.

The second aim of this study was to evaluate the additional value of PSI as compared to other surgical methods. Both the tibia and femur were considered in this study to ensure that our findings would apply to the distinct shapes of these bones (i.e., the round shape of the femur *versus* the triangular prism shape of the tibia). All methods used 3D visualization prior to surgery. While this step requires some time investment, it provides crucial information about all degrees of correction needed for proper postoperative bone alignment. 3D visualization is essential for accurately assessing malunion in all three planes: varus/valgus angulation, ante/recurvation, and rotation. This level of essential assessment is not feasible using 2D radiographs or CT slices alone, recommending 3D analysis for thorough preoperative planning of multiplanar long bone corrective osteotomies. Using merely this technique (e.g. ‘3D visualization’ group) means that during surgery everything was done ‘free-hand’ using temporary k-wires, a goniometer, and the surgeon’s visual estimation, preventing the VSP from being translated into the surgery room with personalised instrumentation (i.e. guides and implants). Given the three-dimensional nature of the required correction, maintaining the final preferred position of the distal bone segment proved challenging, resulting in inaccurate results (as visualised in Fig. 3). During the experimental procedures, fluoroscopy imaging techniques were employed to determine the osteotomy plane by measuring from the knee joint, but this approach led to higher plane errors (e.g., 6.3 mm error for ‘3D visualization’ versus 3.2 mm and 1.4 mm for ‘3D guided’ and ‘3D PSI’, respectively) due to the lack of a surgical osteotomy guide. Adding these surgical guides into the 3D visualization process translated the 3D planning data to the operating room, allowing for easier determination of the osteotomy plane. However, when a contoured “off-the-shelf” implant was used (i.e., the ‘3D guided’ group), it sometimes forced the bone back into a suboptimal position during fixation when screws were applied, as seen in our results when comparing it to PSIs. If a conventional implant fits the corrected bone shape, potentially by contouring, this technique could be applied and may achieve accurate corrections as showcased in prior studies [9, 23]. However, contouring often leads to a suboptimal fit and alters the implant’s mechanical properties, which must be taken into account. Especially in the lower extremities as loadbearing puts more strain to these constructs. In complex shaft corrective osteotomies of the long bones, particularly those requiring multiplanar corrections, in combination to the largely altered bone anatomy (i.e. due to extensive callus formation, comminution or displaced bone fragments at the time of injury) may prevent a conventional implant from fitting properly. In such cases, replacing the conventional implant with a PSI (e.g. the ‘3D PSI’ group) offers advantages. With a PSI, the implant itself guides the bone fragments into the desired position, independent of the underlying bone structure, thus eliminating the sources of loss of correction due to improper conventional implant fitting present in the ‘3D guided’ group. Initial results using merely three specimens indicated that 3D PSI resulted in the most accurate outcomes of the three methods (see Fig. 5). Additionally, the unique challenges of the anterior crest and triangular shape of the tibial shaft were no issue for the PSIs, which was particularly successful in maintaining rotational correction (e.g. 3.5 ⁰ for ‘3D PSI’ versus 11.9 ⁰ and 18.7 ⁰ for ‘3D visualisation’ and ‘3D guided’, respectively for all rotational angles). This is critical for managing the step formation that can occur between bone segments during rotation, a problem that is difficult to resolve with conventional implants.

This study has several limitations. First, all specimens contained no previous deformities and thus led to the use of reverse deformities to reproduce osteotomy procedures. The workflow included severe reversed osteotomies with rotations in three dimensions, to simulate complex corrective osteotomy surgery. The absence of malunions additionally resulted in smooth bone structures, no abnormal bone growth (i.e. irregular surfaces due to pervious fractures) was present. This abnormal bone growth can normally complicate the fit of conventional implants during treatments of malunions, but was not possible to consider in the current study. Using reversed corrective osteotomies of normal cadaveric specimens, was the only way to compare three methods directly, including using free-hand techniques during surgery opposed to 3D technology instruments, which was not done in previous works [18, 24, 29]. Secondly, although the number of specimens available for this study was limited, it was sufficient to conduct this proof-of-principle study on the use of PSIs in multiplanar long bone corrective osteotomy surgeries. Thirdly, using a 3D workflow and creating patient-specific surgical guides or implants involves additional financial investment and manpower. We acknowledge that the hourly rate of technical staff required to prepare the 3D workflow and designing 3D parts, along with the 3D printing costs for guides and the design and fabrication of implants, should be considered. These costs can vary depending on the team’s expertise and geographical location. Currently, the exact cost of producing these implants remains uncertain, as they are being developed in a research setting. However, we believe these costs are not excessive and that the benefits of 3D technology for these types of surgeries could potentially result in significant cost savings in the operating room. Possible advantages include reduced surgical time, a minimized risk of complications, and a lower likelihood of revision surgeries, all of which could offset the initial expenses. Nonetheless, for broader implementation of these solutions in patient care, a comprehensive cost-effectiveness analysis would be essential to fully evaluate and understand their economic impact. Lastly, the focus of this study was merely on postoperative accuracy measurements in a cadaveric setting, thus the costs and time investments, radiation exposure, surgery time and postoperative patient reported outcome were not part of current in vitro study. A prospective clinical trial is recommended to fully assess the additional value of the use of PSIs in corrective osteotomy surgery.

In conclusion, the use of patient-specific plates combined with 3D-printed drilling guides for complex multiplanar corrective osteotomies of femoral and tibial shaft malunions is feasible and results in accurate reconstructions, in particular in the axial plane to correct rotational malalignment. This innovative technique allows precise determination of the osteotomy plane, guides the intended correction, and maintains the correction with a perfectly fitting implant, translating the surgical plan accurately into the procedure. The 3D PSI method offers particular benefits in complex long bone corrective osteotomies where bone anatomy significantly deviates from the norm due to factors such as extensive callus formation, comminution, or displaced bone fragments. Future research should focus on applying PSIs combined with 3D-printed guides in clinical settings to investigate their performance in actual malunion cases rather than simulated deformities.

### Appendices

Appendix 1: Postoperative evaluation of the accuracy of femoral shaft corrective osteotomies.

**Table:** 

	Side	Valgus/varus (◦)	Ante/recurvation (◦)	Rotation (◦)	Osteotomy plane error (mm)
3D visualization	Right	8.7	3.3	17.8	5.2
	Left	1.6	11.2	13.1	8.7
	Median	5.2	7.2	15.4	7.0
3D guided	Right	6.6	2.6	22.8	14.5
	Left	8.5	16.1	4.6	4.1
	Median	7.5	9.3	13.7	9.3
3D PSI	Right	2.8	1.3	11.8	3.5
	Left	2.3	1.1	4.3	0.4
	Median	2.5	1.2	8.0	1.9

Accuracy was assessed in terms differences between the 3D virtual surgical plan and the postoperative obtained position considering varus/valgus angulation (degrees), ante/recurvation (degrees), rotation (degrees), and osteotomy plane error (millimeters)

Appendix 2: Postoperative evaluation of the tibial corrective shaft osteotomies.

**Table:** 

	Side	Valgus/varus (◦)	Ante/recurvation (◦)	Rotation (◦)	Osteotomy plane error (mm)
3D visualization	Right	2.3	1.0	10.8	4.8
	Left	4.4	4.2	9.4	7.1
	Median	3.4	2.6	10.1	6.0
3D guided	Right	2.2	1.9	24.0	2.2
	Left	2.6	5.0	14.5	0.3
	Median	2.4	3.4	19.2	1.3
3D PSI	Right	2.1	0.5	2.6	1.0
	Left	1.7	2.4	1.2	1.9
	Median	1.9	1.4	1.9	1.4

Accuracy was assessed in terms of differences between the 3D virtual surgical plan and the postoperative obtained position considering varus/valgus angulation (degrees), ante/recurvation (degrees), rotation (degrees), and osteotomy plane error (millimeters)

## Data Availability

No datasets were generated or analysed during the current study.
